# Drosophila HP1c Is Regulated by an Auto-Regulatory Feedback Loop through Its Binding Partner Woc

**DOI:** 10.1371/journal.pone.0005089

**Published:** 2009-04-07

**Authors:** Jochen Abel, Ragnhild Eskeland, Grazia D. Raffa, Elisabeth Kremmer, Axel Imhof

**Affiliations:** 1 Munich Centre of Integrated Protein Science and Adolf-Butenandt Institute, Ludwig Maximilians University of Munich, Munich, Germany; 2 Dipartimento di Genetica e Biologia Molecolare Universita' di Roma La Sapienza, Piazzale Aldo Moro, Rome, Italy; 3 Helmholtz Zentrum München, Institute of Molecular Immunology, Munich, Germany; National Institute on Aging, National Institutes of Health, United States of America

## Abstract

HP1 is a major component of chromatin and regulates gene expression through its binding to methylated histone H3. Most eukaryotes express at least three isoforms of HP1 with similar domain architecture. However, despite the common specificity for methylated histone H3, the three HP1 isoforms bind to different regions of the genome. Most of the studies so far focused on the HP1a isoform and its role in transcriptional regulation. As HP1a requires additional factors to bind methylated chromatin *in vitro*, we wondered whether another isoform might also require additional targeting factors. Indeed, we found that HP1c interacts with the DNA binding factors Woc and Row and requires Woc to become targeted to chromatin *in vivo*. Moreover, we show that the interaction between HP1c and Woc constitutes a transcriptional feedback loop that operates to balance the concentration of HP1c within the cell. This regulation may prevent HP1c from binding to methylated heterochromatin.

## Introduction

Most eukaryotes have at least three HP1 isoforms [1], which are conserved in overall structure but bind to different regions within the genome [2]. In addition to the three ubiquitously expressed isoforms, HP1d/rhino and HP1e have been described in *Drosophila* to be primarily expressed in germ cells [3]. The chromo domain of various HP1 isoforms interacts specifically with H3 molecules methylated at lysine 9 [4]–[7]. As this modification is mostly found in transcriptionally inactive or repressed regions [8], [9], HP1a is mostly considered to have a repressive function. This is further substantiated by experiments in which HP1 has artificially been targeted to an integrated promoter [10]. However, this view of HP1a acting merely as a repressor has been challenged by the fact that some heterochromatic genes require HP1 for active transcription [11], [12]. Knock down experiments targeting HP1a revealed that a considerable fraction of genes were down-regulated by HP1a arguing for an activating rather than a repressing role of this protein [13]. Even more strikingly HP1a gets targeted to highly expressed genes such as hsp70 thereby regulating its expression after heat shock [14]. Besides its ambiguous function in gene regulation, the role of histone methylation as the primary targeting function has been challenged recently. RNA does for example also play an important targeting function via its binding to the hinge region of the mammalian isoform of HP1a [15]. In fact when the hinge region is mutated, HP1 can no longer bind to chromatin in an *in vitro* binding assay [16]. The involvement of RNA in targeting HP1 to its binding-site within the genome is also evident in *S. pombe*, where the recruitment of the yeast HP1 orthologue SWI6 is dependent on the generation of short double stranded RNAs from heterochromatic loci [17], [18]. Another important factor of targeting HP1 to its cognate binding site is the interaction with known chromatin associated factors such as Su(var)3–9 or ACF1 [19]. The hypothesis that multiple interactors mediate HP1 binding to chromatin is further substantiated by experiments that show the importance of the hinge and the chromo shadow domain for the differential targeting of specific isoforms [20], [21]. The canonical HP1a isoform has been shown to interact with a multitude of different interactors [22] explaining many of the functions that HP1 fulfills *in vivo*.

### HP1 variants

Despite the wealth of information that has been accumulated for HP1a, the information on the other HP1 variants is sparse. In case of the mammalian HP1 beta and gamma, only few specific interactors have been reported. It has been suggested that HP1 beta and gamma can exchange at a given binding site depending on the physiological status of the cell or the activity state of the promoter [23]. Like for HP1a the other HP1 isoforms can act as transcriptional repressors [24]–[26] or activators [27], [28] depending on promoter architecture. Considering the fact that all isoforms contain a chromo domain and have the ability to bind methylated histone H3, it is surprising that the different isoforms bind to vastly different regions of the genome [2], [21]. We therefore wondered whether the differential targeting is dependent on the interaction of different HP1 isoforms with various cofactors. By performing an affinity purification using epitope-tagged HP1c we indeed found the euchromatic isoform of HP1 in Drosophila, HP1c, binds to two Zn-finger containing DNA binding factors, Woc and Row [28], which is in marked contrast to HP1a, which does not interact with these proteins. This finding led us to speculate that the levels of HP1 isoforms have to be tightly balanced with that of their binding partners in order to prevent an interference, which could severely disturb chromatin structure. In fact it has been recently shown that a proper balance of the two HP1 isoforms in *S. pombe* is strictly required for the establishment and maintenance of pericentric heterochromatin [29]. Indeed, when we analysed the effect of Woc on targeting and expression of HP1c we found a strong interdependence. Woc acts as a transcriptional activator for HP1c's expression. HP1c in contrast impairs the ability of Woc to stimulate transcription from the endogenous HP1c locus, thereby generating a negative feedback loop that ensures a balanced level of Woc and HP1c *in vivo*.

## Results and Discussion

**Figure 1:**
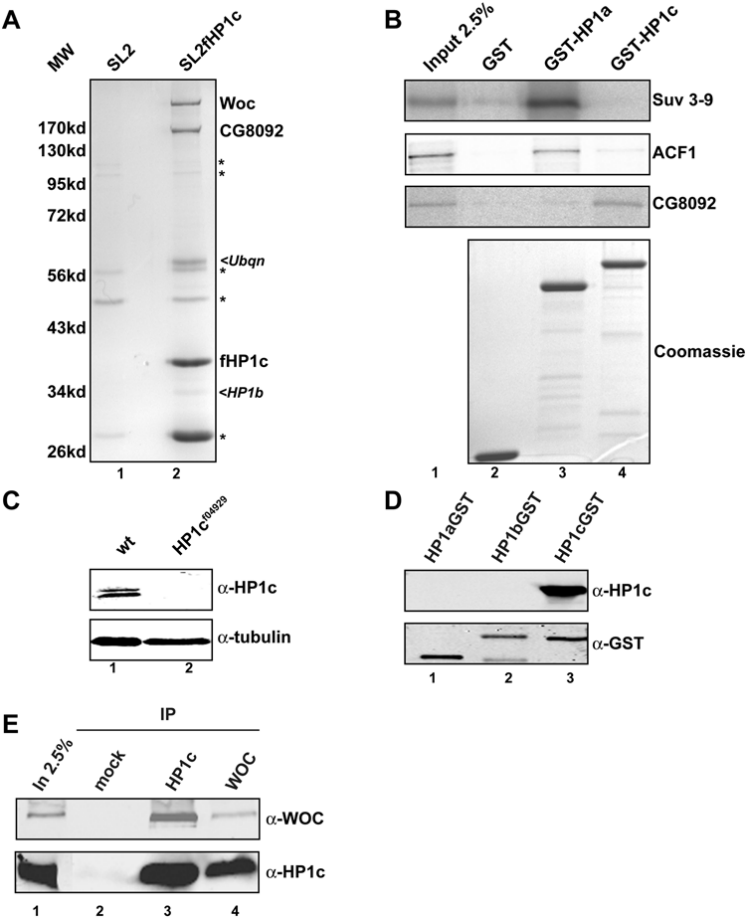
HP1c interacts with two Zn-finger containing proteins Woc and ROW. (A) Comassie staining of a flag affinity purification from nuclear extracts of SL2 cells (lane 1) or SL2 cells stably transfected with an expression vector for HP1c (lane 2). Major proteins are indicated in bold letters, proteins that were co-purified to a various degree in different preps are indicated in italics. Signals derived from the M2-antibody or proteins that are also present in the controlare indicated with an asterisk. (B) GST pull down assays using GST (lane 2), GST-HP1a (lane 3) or GST-HP1c (lane 4) as a bait and in vitro translated Su(var)3–9 (top panel) ACF1 (second panel) or ROW (bottom panel) as prey (2.5% of the input material is shown in lane 1). To ensure equal loading the SDS-PAA gel was stained with coomassie blue (bottom panel). (C) Specificity of the HP1c antibody. Western Blot on purified recombinant HP1 isoforms using the monoclonal HP1c antibody used in this study (top panel) and an anti-GST antibody (bottom panel). (D) Whole extract of wt or HP1c−/− mutant flies were subjected to SDS-PAGE and blotted using an anti HP1c (top panel) or an anti tubulin antibody. (E) Immunoprecipitation assays using nuclear extracts of early Drosophila embryos (0–12 h). Co-precipitated proteins were detected by Western Blotting. A mock immunoprecipitation using a non specific antibody was performed as a control (lane 2).

HP1a requires additional factors to get targeted to H3K9methylated chromatin [19]; we thus wondered which factors interact with the highly related HP1c protein. We immunoprecipitated FLAG tagged HP1c (fHP1c) from Drosophila SL2 cells and purified a protein complex containing fHP1c and two cofactors Woc and Row (Figure 1A), which is in agreement of the results from Font-Burgada and colleagues [28]. All three proteins were indentified by GeLC MS/MS mass spectrometry and LC MS/MS analysis of the eluted protein complex in at least two independent protein purifications. Besides the three major stoichiometric components HP1c, Row and Woc that were identified with MOWSE scores of (274, 335 and 119) we also found varying amounts of Ubiquilin, HP1b and eIF4a, the functional significance of which has not been further studied. Both major HP1c associated proteins (Woc and Row) contain several Zn-finger domains and two or three HMG-I/Y domains, respectively. In the case of Woc three mammalian orthologues (ZNF198, ZNF261 and ZNF 262) [30]–[32] have been reported that are mutated in myeloproliferative diseases (ZNF198) or X-linked mental retardation (ZNF 261). In *Drosophila*, Woc is involved in regulating genes that are crucial for ecdysone biosynthesis [33], [34] and prevents telomeric fusions [35]. The other Zn-finger protein, Row, is poorly characterized but has recently been shown to co-regulate certain neuronal genes together with Woc and HP1c [28].

### HP1 isoforms bind selectively to different protein partners

Interestingly, the HP1c complex we purified does not contain either Su(var)3–9 or ACF1, two factors that mediate HP1a recruitment to chromatin, suggesting that the HP1c isoform might require a different set of interaction partners for its function. To determine whether HP1a and HP1c form two different complexes with exclusive partners, we expressed HP1a and HP1c as GST fusion proteins and performed GST-pull down experiments using *in vitro* translated Su(var)3–9 or ACF1 (Figure 1B). Whereas HP1a efficiently precipitated these proteins, HP1c did not. In order to test whether the binding of Row or Woc to HP1c is as exclusive as the binding of ACF1 and Su(var)3–9 to HP1a, we tested the *in vitro* translated Woc and Row proteins in a pull down assay (Figure 1B and data not shown). The pull down assay demonstrates that Row specifically interacts with HP1c but not with HP1a (Figure 1B), suggesting a possible role for Row and/or Woc for the specific targeting of HP1c to eukaryotic regions. Interestingly we could not observe an interaction between Woc and HP1c *in vitro* neither when it was expressed separately or together with Row (data not shown). This may be due to an improper folding of *in vitro* translated Woc or a requirement for specific posttranslational modifications that do not occur during in vitro translation and bacterial expression. Alternatively, Woc may require a specific structural arrangement of the complex similarly to the human orthologue of Woc (ZNF198), which has recently been shown to interact with more stably with a trimeric CoRest complex than with the individual subunits [36].

### HP1c interacts with Woc *in vivo*

In order to confirm the specificity of the Woc/HP1c interaction *in vivo*, we developed an HP1c specific monoclonal antibody. This antibody recognizes a protein of the expected molecular weight in extracts from wild-type flies, that is absent in extracts prepared from HP1c−/− strains (Figure 1C) and does not recognize any of the other HP1 isoforms (Figure 1D). Using this antibody we could co-immunoprecipitate Woc from a nuclear extract prepared from 0–12 hr old *Drosophila* embryos (Figure 1E). We also used an anti-Woc antibody for immunoprecipitation, which resulted in the co-purification of HP1c (Figure 1E). Based on these experiments we concluded that most HP1c is associated with two Zn-finger proteins Woc and Row, which do not interact with HP1a.

**Figure 2:**
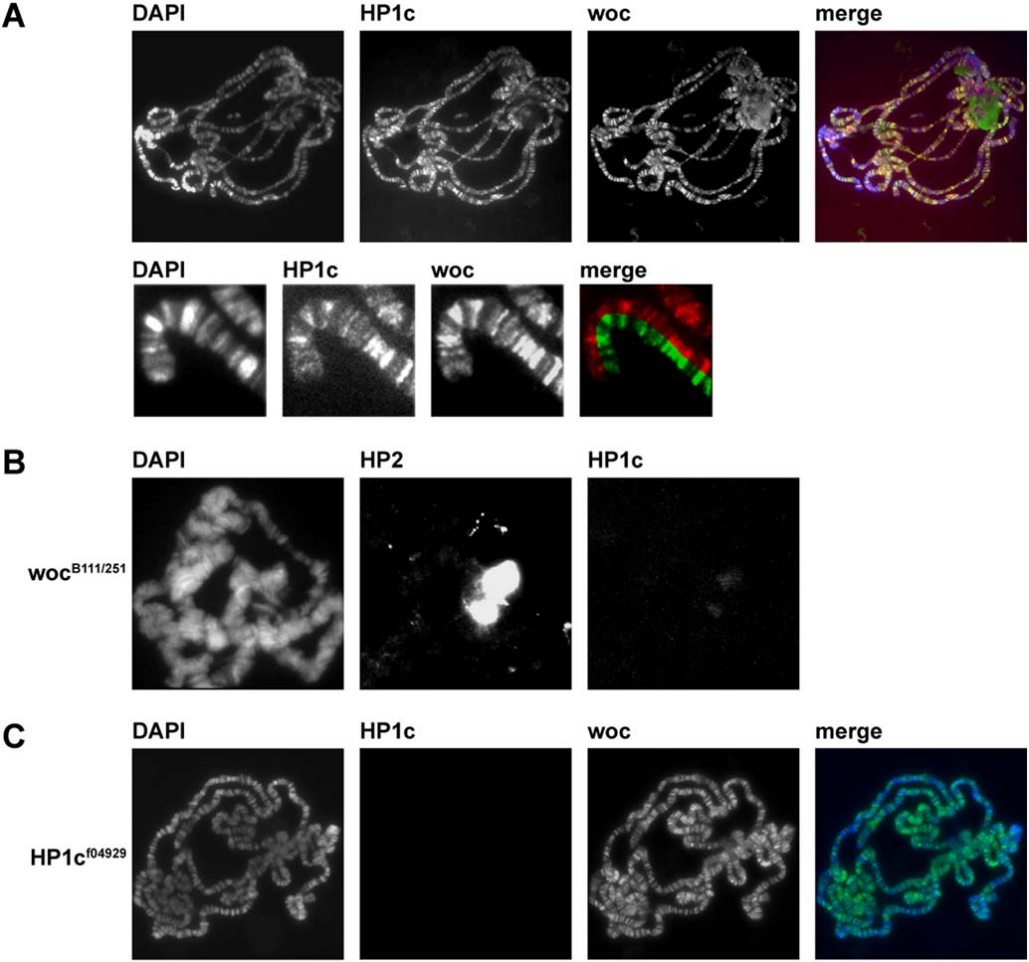
HP1c colocalizes with Woc on polytene chromosomes. (A) Salivary gland polytene chromosomes from wild type larvae stained with α-HP1c and α-woc (upper panel). Enlargement and generation of split images allows a detailed analysis of HP1c and woc localization (lower panel). (B) Salivary gland polytene chromosomes from woc-mutant larvae stained with α-HP1c and α-HP2 as a control. (C) Salivary gland polytene chromosomes from HP1c-mutant larvae stained with α-HP1c and α-woc. In the merged images, woc is depicted in green, HP1c in red. DNA was stained with DAPI (blue).

**Figure 3:**
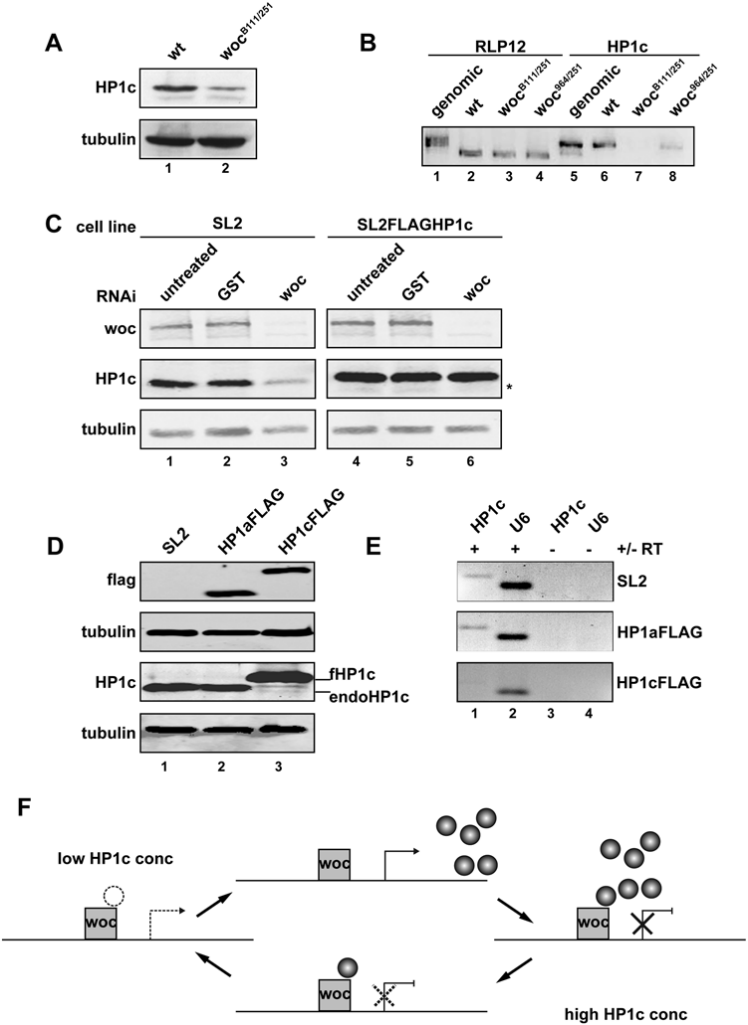
Woc and HP1c coordinate HP1c expression on a transcriptional level. (A) Western blot using whole cell extracts of either wild type or woc-mutant Drosophila 3^rd^ instar larvae. (B) RT-PCR analysis using total RNA isolated from 3^rd^ instar larvae. HP1c expression analysis was performed with wild type larvae and larvae from two fly strains carrying different heteroallelic combinations of Woc mutant alleles. Primers for the ribosomal protein RLP12 spanning an intron were used as a control. (C) Drosophila SL2 cells transfected with (right panel) or without (left panel) an expression construct for a FLAG-tagged version of HP1c driven by an actin promoter were subjected to woc RNAi. Protein levels were determined by Western Blotting with the indicated antibodies. The asterisks indicates the endogenous HP1c (D) Whole cell extracts from *Drosphila* SL2 cells transfected with either HP1aFLAG or HP1cFLAG were prepared. Endogenous HP1c levels were determined by immunoblotting with an HP1c specific antibody (lower panel). Expression of the FLAG-tagged HP1 isoforms was determined as a control. (E) RT-PCR analysis of total RNA using a primer pair specific for endogenous HP1c. RNA was isolated from Drosophila SL2 cells that were either non-transfected or transfected with the indicated expression plasmids. (F) Model of HP1c action to modulate its own transcription by counteracting Woc mediated transcriptional activation.

Using the highly specific antibody we investigated the distribution of HP1c within chromatin. In agreement with previous reports for the mammalian isoforms and for Drosophila Kc cells [21], we found that HP1c is excluded from DAPI dense regions within the nuclei of SL2 cells (data not shown), To map the sites of HP1c binding more precisely we used polytene chromosomes prepared from *Drosophila* third instar larvae. Staining of polytenes showed a strong localization of HP1c to interbands, which are considered to be sites of actively transcribed chromatin (Figure 2A). This is in marked contrast to known heterochromatic proteins such as HP1a or HP2 (Figure 2B), which are highly enriched in pericentric heterochromatin. This is of particular interest as Woc has also been shown to bind to interbands of polytene chromosomes [35]. Indeed, when we performed a co staining of HP1c and Woc we found an almost perfect overlap of the two signals (Figure 2A, merge and details) suggesting that the two proteins indeed form a complex on chromatin. We next tested whether the binding of HP1c to chromatin is dependent on the presence of Woc and vice versa. In order to do this, we prepared polytene chromosomes from HP1c−/− third instar larvae and from a fly strain carrying a heteroallelic combination of *woc* alleles that result in greatly reduced Woc levels (Figure 2B and 2C and [35]). Whereas HP1c mutations did not have a strong effect on Woc binding, mutations in *woc* almost completely abolished HP1c binding. However, this is only in part due to a lack of targeting as the reduction of Woc levels also results in decreased HP1c (but not HP1a) levels (Figure 3A and data not shown). As it has been observed that the reduction of one component of a multi-protein complex destabilizes the other [37], we wondered whether the reduction is indeed due to a decrease in HP1c stability or if the transcription of HP1c is reduced. Thus we performed RT-PCR analysis using total RNA isolated from salivary glands of 3^rd^ instar larvae. We observed a strong reduction of HP1c mRNA in two different *woc* heteroallelic mutant backgrounds, suggesting that besides being a binding partner for Woc, HP1c is also transcriptionally regulated by Woc (Figure 3B). We can not exclude the possibility that the observed effect is indirect but based on the extensive co-localization of HP1c and Woc and the mapping of HP1c to its own genomic locus [2] this seems to be very unlikely.

### HP1c and Woc are parts of an autoregulatory loop to modulate HP1c expression

To further investigate the dynamics of the regulation of the HP1c transcript, we treated SL2 cells either with a *woc* specific dsRNA that efficiently depletes Woc protein (Figure 3C) or an unrelated gene (GST) as a negative control. Whereas the levels of HP1c did not decrease on the negative control, a considerable drop in HP1c levels were observed in the cells treated with dsRNA against Woc (Figure 3C). This was dependent on the endogenous HP1c promoter as the removal of Woc did not lower the amount of exogenous, FLAG tagged HP1c transcribed from an actin promoter (Figure 3C). HP1c expression from an exogenous actin promoter on the contrary completely abolishes expression of the endogenous non-tagged HP1c (Figure 3D) whereas the expression of the HP1a isoform has no effect on HP1c expression. This repression can also be observed on the transcriptional level as exogenous HP1c expression leads to a considerable reduction of the levels of endogenous HP1c mRNA (Figure 3E). Based on these observations, we argue that woc and HP1c can act as antagonistic factors regulating transcription, leading to a simple way of regulating HP1c levels within a cell by a negative feedback loop (Figure 3F). Unfortunately the anti Woc antiserum did not allow us to perform ChIp experiments to show a direct binding of woc to the HP1c promoter. Therefore we do not know whether the effect we see is direct or indirect. However, as Greil and colleagues showed a binding of HP1c to the HP1c locus by Dam-ID [2] and we observe an almost full overlap of Woc and HP1 binding in polytene chromosomes, we would suggest that HP1c as well as its binding partner Woc play a direct role on this locus.

The mechanism by which HP1c inhibits the ability of Woc to activate transcription from the HP1c promoter is unclear. In theory, it could either interfere with the interaction between Woc and DNA or between Woc and transcriptional co-activators. As HP1c and Woc co-localize on polytene chromosomes and we do not observe an effect of HP1c deletion on Woc localization we consider the first model as improbable. As the human orthologue of Woc, ZNF198, interacts with a series of transcriptional regulators [36] we would suggest that the *Drosophila* Woc protein can also bind to such transcriptional cofactors in an HP1c regulated manner. However, additional experiments will be required to dissect the precise molecular function of transcriptional regulation mediated by Woc and HP1c.

## Materials and Methods

### Plasmids and cloning

pFLC-1 ROW, pFLC-1 Woc and HP1c pOT2 were obtained from the Berkeley Drosphila Genome Project (BDGP). HP1a and HP1c were PCR-cloned into pGEX4T1 vectors via XmaI and XhoI and into a pbackFLAG vector via KpnI and SacI. Cloning details are available on request.

### *In vitro* transcription and translations

Cell-free coupled transcription-translations were performed according to the manufacturers instruction (TnT- Quick Kit, Promega). Proteins were transcribed from 1 µg pET15 (Su(var)3–9) or pFLC-1 vectors (WOC, ROW) and translated in the presence of S^35^-Methionine/S^35^-Cysteine mix.

### GST pulldown assays

Approximately 3 µg of GST or the appropriate HP1-GST fusion protein were coupled to glutathione sepharose beads. After extensive washing using CB300 (25 mMTris-Cl pH 7.6, 300 mMNaCl, 0.5 mMEGTA, 10% glycerol, 1 mMDTT, 0.2 mMPMSF), the beads were incubated with *in vitro* translated S^35^-labeled Su(var)3–9, Woc or Row in the presence of ethidium bromide (25 ng/µl). Unbound material was removed by washing with CB200. Bound proteins were eluted with SDS-sample buffer, separated by SDS-PAGE and analysed by autoradiography.

### Complex purification

*Drosophila* SL2 expressing HP1cFLAG were generated by stable transfection with pbackFLAGHP1c. Nuclear extracts from this cell line as well as from non-transfected cells were prepared as previously described [38]. Nuclear extract from approx. 5×10^8^ cells was incubated with M2 anti-FLAG agarose beads (Sigma) for 2 hours at 4°C. After extensive washing with CB300, bound complexes were eluted with FLAG-peptide and separated by SDS-PAGE. Specific interactors were identified by mass spectrometry.

### Immunoprecipitation assays

For co-immunoprecipitation experiments, 30 µl of extracts from early *Drosophila* embryos (TRAX, [39]) were incubated overnight at 4°C with α-HP1c (2G2 subtype IgG2A), α-Woc or buffer (mock IP) in a total volume of 400 µl of BC200. Complexes were immunoprecipitated using protein A/G sepharose. After extensive washing with BC300 bound proteins were eluted with SDS-sample buffer and analyzed by western blotting.

### Immunofluorescence

Polytene chromosomes from the salivary glands of 3^rd^ instar larvae were dissected in 0.7% NaCl and fixed for 8 minutes with 1.85% formaldehyde in 45% acetic acid. Chromosomes were incubated with monoclonal rat α-HP1c and polyclonal rabbit α-Woc at 4°C overnight, followed by incubation with the appropriate Cy3- or AlexaFluor 488-conjugated secondary antibodies. DNA was visualized by DAPI-staining.

### RT-PCR

Total RNA was isolated from both *Drosophila* SL2 cells and from cells transfected with plasmids encoding for FLAG-tagged HP1c. Purfied RNA was reverse transcribed using M-MuLV Reverse Transcriptase (Fermentas) and RT-PCR products were subsequently PCR-amplified and resolved on 2% agarose gels containing ethidiumbromide. In order to distinguish between endogenous and exogenous HP1c-transcripts, a forward primer was used that only anneals to the 5′-UTR sequence of the endogenous transcript, but not to the exogenously derived transcript.
